# Percentage amplitude of fluctuation and structural covariance changes of subjective cognitive decline in patients: A multimodal imaging study

**DOI:** 10.3389/fnins.2022.888174

**Published:** 2022-07-22

**Authors:** Ke Xu, Yichen Wei, Shuming Zhang, Lihua Zhao, Bowen Geng, Wei Mai, Pengyu Li, Lingyan Liang, Duoli Chen, Xiao Zeng, Demao Deng, Peng Liu

**Affiliations:** ^1^School of Life Sciences and Technology, Life Science Research Center, Xidian University, Xi’an, China; ^2^School of Life Sciences and Technology, Engineering Research Center of Molecular and Neuro Imaging Ministry of Education, Xidian University, Xi’an, China; ^3^Department of Radiology, The People’s Hospital of Guangxi Zhuang Autonomous Region, Nanning, China; ^4^Department of Acupuncture, First Affiliated Hospital, Guangxi University of Chinese Medicine, Nanning, China

**Keywords:** subjective cognitive decline, percent amplitude of fluctuation, structural covariance, gray matter volume, magnetic resonance imaging

## Abstract

**Back ground:**

Subjective cognitive decline (SCD) may be the first clinical sign of Alzheimer’s disease (AD). The possible neural mechanisms of SCD are not well known. This study aimed to compare percent amplitude of fluctuation (PerAF) and structural covariance patterns in patients with SCD and healthy controls (HCs).

**Methods:**

We enrolled 53 patients with SCD and 65 HCs. Resting-state functional magnetic resonance imaging (MRI) data and T1-weighted anatomical brain 3.0-T MRI scans were collected. The PerAF approach was applied to distinguish altered brain functions between the two groups. A whole-brain voxel-based morphometry analysis was performed, and all significant regions were selected as regions of interest (ROIs) for the structural covariance analysis. Statistical analysis was performed using two-sample *t*-tests, and multiple regressions were applied to examine the relationships between neuroimaging findings and clinical symptoms.

**Results:**

Functional MRI results revealed significantly increased PerAF including the right hippocampus (HIPP) and right thalamus (THA) in patients with SCD relative to HCs. Gray matter volume (GMV) results demonstrated decreased GMV in the bilateral ventrolateral prefrontal cortex (vlPFC) and right insula in patients with SCD relative to HCs. Taking these three areas including the bilateral vlPFC and right insula as ROIs, differences were observed in the structural covariance of the ROIs with several regions between the two groups. Additionally, significant correlations were observed between neuroimaging findings and clinical symptoms.

**Conclusion:**

Our study investigated the abnormal PerAF and structural covariance patterns in patients with SCD, which might provide new insights into the pathological mechanisms of SCD.

## Introduction

Subjective cognitive decline (SCD) is a condition characterized by self-perceived cognitive decline but is difficult to be detected using objective tests. SCD signifies an intermediate state between normal cognition and mild cognitive impairment (MCI). Although SCD in older adults may function normally, perceived worsening may indicate incipient dementia and predict future deterioration (Viviano and Damoiseaux, 2020). Increasing evidence has suggested that patients with SCD exhibit a trend toward a greater risk of cognitive decline and the development of MCI and Alzheimer’s disease (AD; Jessen et al., 2014). The inclusion of the SCD concept completes the full picture of AD development (from normal state to SCD to MCI and AD) and provides the basis for the diagnosis and intervention of AD at the very early stage of the disease (Jessen et al., 2014; Si et al., 2020).

Previous brain imaging studies have reported structural and functional changes in patients with SCD. For example, several structural magnetic resonance imaging (MRI) studies have indicated that patients with SCD have a loss of gray matter volume (GMV) in the hippocampus (van der Flier et al., 2004; Saykin et al., 2006), and frontotemporal regions (Saykin et al., 2006). In voxel-based morphometry (VBM) studies, subjective memory impairment has shown atrophy in the frontal, temporal, and parietal lobes compared with healthy controls (HCs) although it is not as extensive as that in MCI (Hong et al., 2015). Saykin et al. (2006) have reported that, compared to HCs, the level of medial temporal lobes atrophy and frontal lobes atrophy of patients with SCD is related to the degree of cognitive decline. The resting-state functional MRI (rs-fMRI) literature has shown a higher amplitude of low-frequency fluctuation (ALFF) values primarily in the bilateral inferior parietal lobule, right middle occipital gyrus, right inferior occipital gyrus, right superior temporal gyri, and right posterior lobe of the cerebellum in patients with SCD, indicating a possible compensatory mechanism in the early stage of AD (Sun et al., 2016). Another functional imaging study also suggested a greater functional connectivity within the default mode network (DMN) including the bilateral precuneus cortex, bilateral thalamus, and right hippocampal regions in patients with SCD relative to controls (Parker et al., 2020). In a review of functional neuroimaging, Viviano et al. pointed out that elevated functional connectivity in SCD could reflect compensatory intrinsic signaling resulting from gray matter atrophy or other neural insults (Viviano and Damoiseaux, 2020). To some extent, these studies may reveal the abnormal brain neural mechanisms of patients with SCD.

However, ALFF/fractional ALFF (fALFF) in SCD is not very consistent in the previous studies (Sun et al., 2016; Yang et al., 2018; Zhang et al., 2021), and more reliable methods may be needed to study altered spontaneous brain activity in SCD. Percent amplitude of fluctuation (PerAF), as a recent voxel-level amplitude metric, is the percentage of BOLD fluctuations relative to the mean BOLD signal intensity for each time point and averaging across the whole time series (Jia et al., 2020). It has been proven to be more reliable and sensitive than ALFF and fALFF in test-retest reliability analysis. The PerAF approach has been used to explore neural mechanisms of diseases, such as MCI (Yu et al., 2019). But, to our knowledge, the PerAF approach has not been applied to assess SCD, which could be potentially valuable to improve our understanding of SCD. Moreover, structural covariance is an effective method to investigate covary in GMV between different brain regions and it is used to study gray matter (GM) covariance by mapping GM correlations of the whole brain to seed regions (Mechelli et al., 2005; Alexander-Bloch et al., 2013; Evans, 2013). Compared with a single brain area volume, the structural covariance of GM may provide more neural information. For example, the pattern of structural covariance may reflect the effects of brain development or structural plasticity (Mechelli et al., 2005; Butz et al., 2009; Ayyash et al., 2021). In recent years, structural covariance analysis has been used in many studies (Wang et al., 2018; Mazzeo et al., 2019; Liu et al., 2020; Fu et al., 2021; Wu et al., 2021). For instance, the structural covariance network seeded from subfields of the hippocampus in patients with MCI has shown an increased extent of structural association compared with HCs (Wang et al., 2018). In another study of structural covariance patterns (Fu et al., 2021), decreased structural covariance and weakened connectivity strength have been observed in patients with SCD compared with normal controls, but which was using 10 regions involved in high-order cognitive function and AD-related dysfunctional structures as regions of interest (ROIs) without choosing from the perspective of GMV-different brain regions.

In this study, we combined PerAF and VBM methods to investigate group differences in regional spontaneous brain activity and GMV in the whole brain, and to assess ROI-related whole brain structural covariance patterns. We also wanted to explore correlations between the neuroimaging findings and clinical features. We hypothesized that (1) patients with SCD could show increased PerAF compared to HCs; (2) Compared to HCs, patients with SCD could display atrophy in regions associated with cognition including memory, attention, and monitoring processes; (3) patients with patients with SCDand HCs could exhibit distinct patterns of structural covariance, and (4) changes of neuroimaging findings might correlate with cognitive performance in patients with SCD.

## Materials and methods

### Subjects

The present sample included 54 patients with SCD and 65 HCs. Based on the definition of SCD by Jessen et al. (2014), inclusion criteria for SCD were as follows: (1) Right handedness; (2) Aged 55–75 years old; (3) Self-reported cognitive decline; (4) Normal general cognitive examination scores: Montreal Cognitive Assessment (MoCA): primary school and below > 19, secondary school > 22, university > 24; Mini-Mental State Examination (MMSE): illiterate > 17, primary school > 20, junior school and above > 24 points; Clinical Dementia Rating (CDR): 0. Subjects were excluded if they had any of the following: (1) MCI or dementia; (2) Vascular disease; (3) Severe depression; (4) Neurological diseases that may cause cognitive problems (such as brain tumors, Parkinson’s disease, encephalitis, epilepsy, etc.); (5) Brain trauma; (6) Other systemic diseases that can cause cognitive impairment, such as thyroid dysfunction, severe anemia, syphilis, HIV, etc.; (7) People with a history of mental illness or congenital intellectual disability; (8) Severe hearing or visual impairment, language communication disorders; (9) MRI contraindications (e.g., metal dentures or other metal implants that cannot be removed, claustrophobia, etc.); (10) Non-handed elderly people.

### Cognitive function test

All subjects underwent a detailed neuropsychological evaluation. MMSE and MoCA are designed to assess cognitive function. The MMSE evaluates orientation, memory, calculation and attention, recall, and language. MoCA is a brief (about 10-min) screening tool for MCI that evaluates visual space, executive function (clock drawing), naming, attention, language, abstract ability, memory, and orientation with a total score of 0–30. The higher score of MMSE and MoCA represents a better cognitive function. The geriatric depression scale (GDS) is used widely as a screening instrument for depression worldwide.

### Magnetic resonance imaging data acquisition

MRI data were acquired on a 3.0 T Siemens Magnetom Verio MRI System (Siemens Medical, Erlangen, Germany), using a standard head coil. During the scan, all subjects were instructed to keep their eyes closed and stay awake, stay still, and not to think of anything in particular. Foam pillows were used for minimizing movement between the instrument and each subject’s head. Rs-fMRI data were acquired by a single-shot gradient-recalled echo planar imaging (EPI) sequence: repetition time (TR) = 2000 ms; echo time (TE) = 30 ms; flip angle (FA) = 90°; field of view (FOV) = 240 mm × 240 mm; matrix size: 64 × 64; slice thickness = 5 mm (no-gap); 31 slices and 180 volumes. High-resolution T1-weighted images were then obtained with magnetization-prepared rapid acquisition gradient echo sequences (3D MPRAGE) with the following parameters: TR = 1900 ms; TE = 2.22 ms, FOV = 250 mm × 250 mm, matrix size: 256 × 256, FA = 9°, slice thickness = 1 mm and 176 slices.

### Functional magnetic resonance imaging data preprocessing

Based on Statistical Parametric Mapping 12 (SPM12, United Kingdom^1^) on the MATLAB platform, the preprocessing of rs-fMRI imaging data was conducted by the Data Processing Assistant for rs-fMRI (DPARSF^2^) software embedded in Data Processing Analysis of Brain Imaging (DPABI 4.3^3^; Yan et al., 2016). The first 5 volumes of functional data for each subject were discarded for signal equilibrium and subject adaptation to the imaging noise. The remaining volumes were slice-timing corrected and head-motion corrected. After realignment, all images were normalized to the standard Montreal Neurological Institute (MNI) template and then resampled into 3 mm^3^ × 3 mm^3^ × 3 mm^3^ resolution. A total of 24 head motion parameters, average signals from the white matter, cerebrospinal fluid, and global signals were used as nuisance covariates to reduce the effects of head motion and non-neuronal blood oxygenation level-dependent (BOLD) fluctuations. The fMRI data were then spatially smoothed with a Gaussian smoothing kernel (full-width half maximum, FWHM = 6 mm). To reduce low-frequency drift and high-frequency respiratory and heart rhythms, the linear trend in the fMRI data was removed, and the images were temporally bandpass filtered (0.01–0.08 Hz).

### Percent amplitude of fluctuation computation

PerAF of each voxel was estimated with the following equations (Jia et al., 2020):


(1) $$\text{PerAF}=\frac{1}{n}\,\sum_{i=1}^{n}\left|\frac{X_{\text{i}}-\mu }{\mu }\right|\times 100\%$$

$$\mu =\frac{1}{n}\,\sum_{i=1}^{n}X_{\text{i}}$$

Here, “*X”* represents the signal intensity of the time point, “*n*” refers to the total number of time points of time course, and“μ” is the mean value of the time course.

### Structural magnetic resonance imaging data preprocessing

Voxel-based morphometry analysis was performed using CAT12 toolbox based on Statistical Parametric Mapping 12 (SPM12, see text footnote 1). Briefly, T1 images were first segmented into gray matter, white matter, and cerebrospinal fluid by tissue probabilistic maps. The native-space images were normalized to the Montreal Neurological Institute (MNI) space with the diffeomorphic anatomical registration through exponential Lie algebra algorithm by linear and non-linear transformation. All images were resliced to a voxel size of 1.5 mm^3^ × 1.5 mm^3^ × 1.5 mm^3^. After correcting for bias-field inhomogeneities, the gray matter images were smoothened with an 8-mm full-width at half the maximum isotropic Gaussian kernel.

Considering individual differences in brain size, each subject’s total intracranial volume (TIV) was computed and used as a covariate in subsequent statistical analysis.

### Structural covariance computation

Whole-brain structural covariance analyses were performed on the modulated GM images for each group using the general linear model in SPM12. We chose the ROIs from the brain areas that showed significant differences between the groups in the GMV analysis. The mean GMV of each ROI was included in the multiple regression models as a covariate of interest to obtain the group level whole-brain voxel-wise structural covariance of the ROIs.

According to previous studies (Wang et al., 2018; Ge et al., 2020; Zhang et al., 2022), we performed a between-group analysis of structural covariance patterns by calculating the slope differences. The analysis used a multiple classic linear regression:


(2) $$Y=\beta_{0}+\beta_{1}\,X+\beta_{2}\,G\,r\,o\,u\,p+\beta_{3}\,\left(X+\text{Group}\right)+\beta_{4}\,\text{Age}$$

$$+\beta_{5}\,\text{Education}+\beta_{6}\,\text{Gender}+\beta_{7}\,\text{TIV}+\varepsilon$$

In Equation 2, X represents a vector of averaged GMV of ROIs and Y represents a vector of the GMV of each region showing group differences. Group is a vector with binary group labels (with 1 indicating patients with SCD and –1 indicating HCs). Age, education, gender, and TIV were considered nuisance variables. β*_3_* was seen to show significant X × Group interaction.

### Statistical analysis

Differences in demographic and clinical features between patients with SCD and HCs were examined by the chi-square test and two-sample *t*-test using SPSS 22.0 (IBM, Armonk, NY, United States). The threshold for statistical significance was set at the level of *p* < 0.05.

A two-sample *t*-test was aimed to examine PerAF-related and GMV-related differences between patients with SCD and HCs. Statistical results were thresholded at *p* < 0.05 [false discovery rate (FDR) corrected].

One-sample *t*-test were performed to map the voxels that expressed a significant positive GM covariance with each ROI in each group with the inclusion of age, education, gender, and TIV as covariates. The significance level was set at *p* < 0.05 (FDR corrected).

To investigate relationships between imaging results and clinical symptoms and to avoid any unintentional bias induced by region-specific prior hypotheses, we conducted a whole-brain regression analysis to evaluate the relationships of PerAF and GMV with MMSE and MoCA, separately. The significance level was set at *p* < 0.05 (FDR corrected).

## Results

### Demographics and clinical results

Their demographic and neuropsychological data are summarized in Table 1. A total of 118 subjects were enrolled in this study, including 53 patients with SCD and 65 matched HCs. There were no significant differences between the two groups in terms of age, education, gender, TIV, and MoCA score (*p* > 0.05). However, patients with SCD showed decreased MMSE score (*p* < 0.05).

**TABLE 1:** Demographic and clinical characteristics of subjects.

Characteristic	Mean ± SD		*P-value*
	SCD (*n* = 53)	HCs (*n* = 65)	
Age (years)	65.91 ± 5.32	64.83 ± 5.82	0.887^a^
Gender (M/F)	16/37	23/42	0.551*^b^*
Education (years)	11.76 ± 3.06	12.03 ± 3.00	0.504^a^
TIV	1347.86 ± 129.17	1368.42 ± 137.87	0.413^a^
MMSE	28.93 ± 0.87	29.22 ± 0.69	0.046^a^
MoCA	25.40 ± 2.13	26.03 ± 2.01	0.102^a^
GDS	4.85 ± 2.60	4.17 ± 2.47	0.153^a^

^a^p values were calculated with two-sample t-test.

^b^p values were calculated with a chi-square test.

SD, standard deviation; SCD, subjective cognitive decline; HCs, healthy controls; TIV, total intracranial volume; MMSE, Mini-Mental State Examination; MoCA, Montreal Cognitive Assessment; GDS, geriatric depression scale.

### Percent amplitude of fluctuation results

**FIGURE 1:**
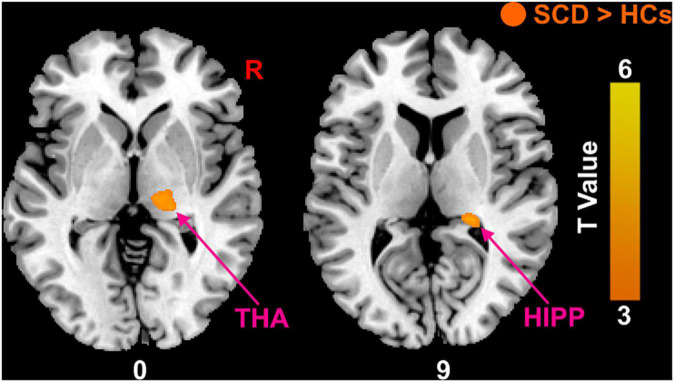
The PerAF differences between subjective cognitive decline (SCD) patients and healthy controls (HCs). Brain regions marked by warm color represent significantly increased PerAF in patiens with SCDs relative to HCs.

**FIGURE 2:**
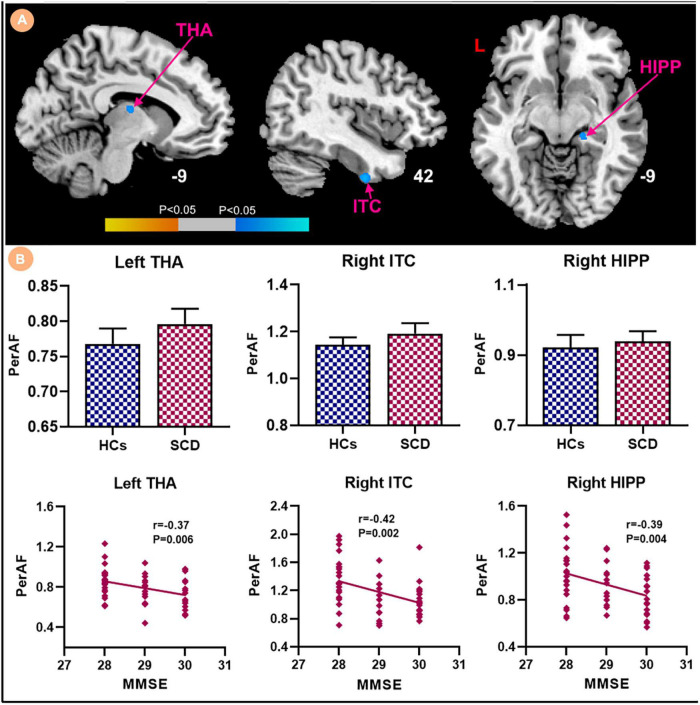
Relationships between the PerAF and MMSE in subjective cognitive decline (SCD) patients. **(A)** PerAF covarying with MMSE in patiens with SCDs. Brain regions marked by warm color represent positive correlation and brain regions marked by cool color represent negative correlation. **(B)** Extracted PerAF values and negative correlations between the MMSE and brain regions including the left THA, right ITC and right HIPP [mean ± standard error of mean (SEM)].

Compared with HCs, the results showed that patients with SCD had increased PerAF in the right hippocampus (HIPP) and right thalamus (THA; Figure 1). Within-group regressions in patients with SCD revealed that there were significant negative correlations between MMSE and PerAF in the right HIPP, right ITC, and left THA (Figure 2).

### Gray matter volume results

**FIGURE 3:**
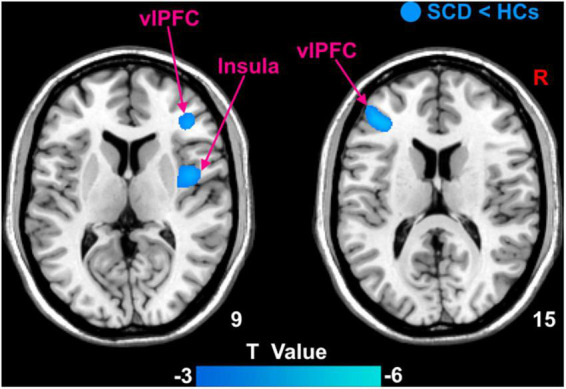
The GMV differences between subjective cognitive decline (SCD) patients and healthy controls (HCs). Brain regions marked by cool color represent significantly decreased GMV in patiens with SCDs relative to HCs.

**FIGURE 4:**
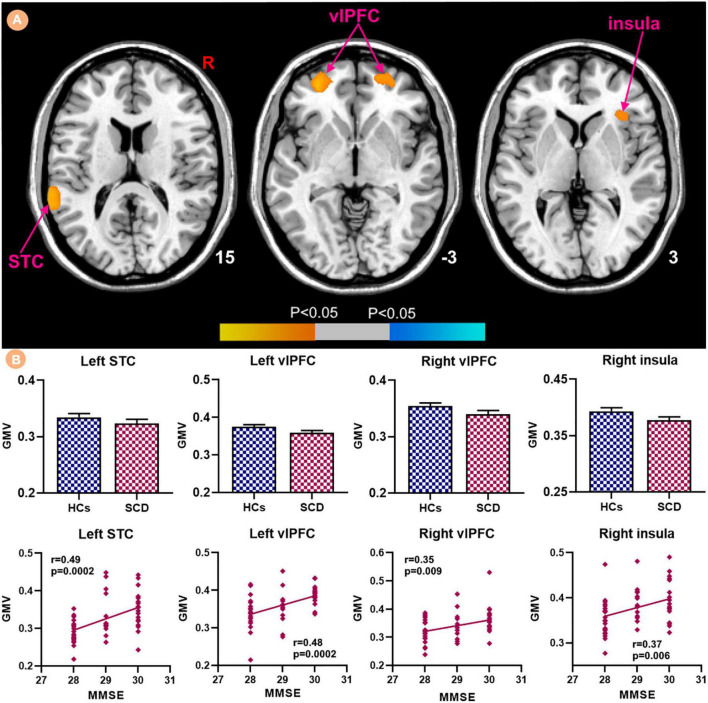
Relationships between the GMV and MMSE in subjective cognitive decline (SCD) patients. **(A)** GMV covarying with MMSE in patiens with SCDs. Brain regions marked by warm color represent positive correlation and brain regions marked by cool color represent negative correlation. **(B)** Extracted GMV values and positive correlations between the MMSE and brain regions including left STC, bilateral vlPFC and right insula [mean ± standard error of mean (SEM)].

Patients with SCD showed decreased GMV in the bilateral ventrolateral prefrontal cortex (vlPFC) and right insula (Figure 3). Within-group regressions in patients with SCD revealed that there were significant positive correlations between MMSE and GMV in the bilateral vlPFC, right insula, and left superior temporal cortex (STC; Figure 4).

### Structural covariance results

**FIGURE 5:**
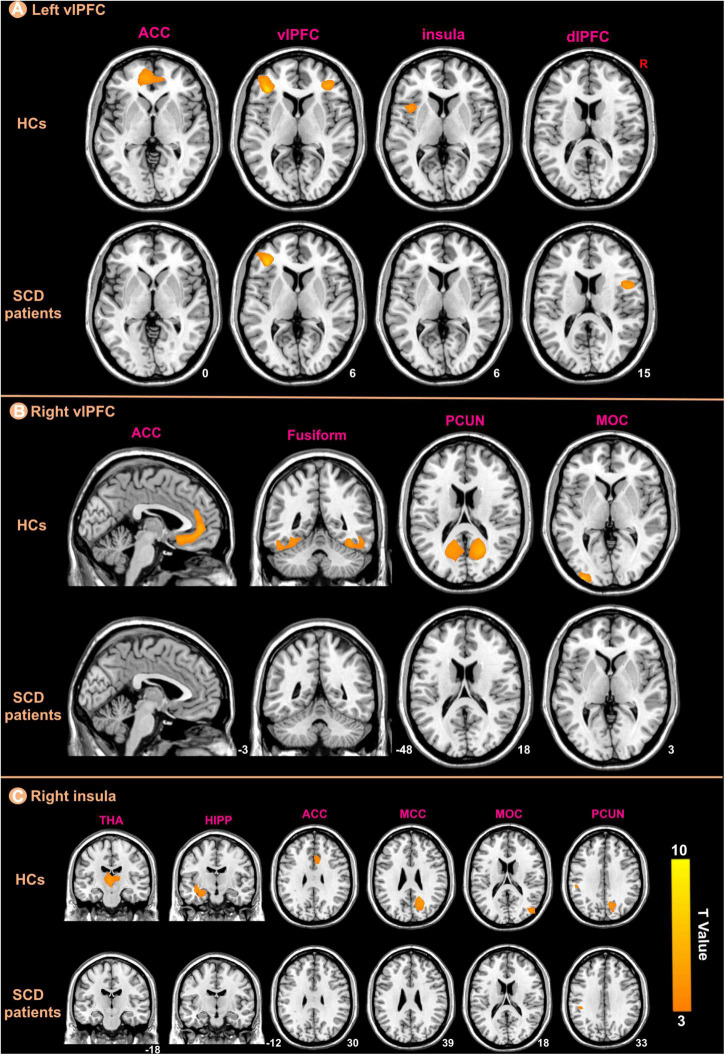
The ROIs-based structural covariance patterns in subjective cognitive decline (SCD) patients and healthy controls (HCs). The structural covariance patterns related with the left vlPFC **(A)**, the right vlPFC **(B)**, and the right insula **(C)** ROIs in the two groups.

We selected three regions including bilateral vlPFC and right insula from those that showed significant differences between the groups in the GMV analysis as ROIs for structural covariance analysis. Figure 5 indicated the different patterns of structural covariance in the two groups associated with the three ROIs and we focused only on the positive correlation. There was an abnormal structural association between the left vlPFC and regions, including the left anterior cingulate cortex (ACC), right vlPFC, left insula, and right dorsolateral prefrontal cortex (dlPFC) in the patients with SCD compared to the HCs. The left ACC, left middle occipital cortex (MOC), bilateral fusiform and bilateral precuneus (PCUN) showed decreased structural covariance with the right vlPFC in patients with SCD compared to HCs. For the right insula ROI, decreased structural covariance was observed between the right insula and regions including the right median cingulate cortex (MCC), right PCUN, right MOC, left HIPP, left THA, and right ACC.

**FIGURE 6:**
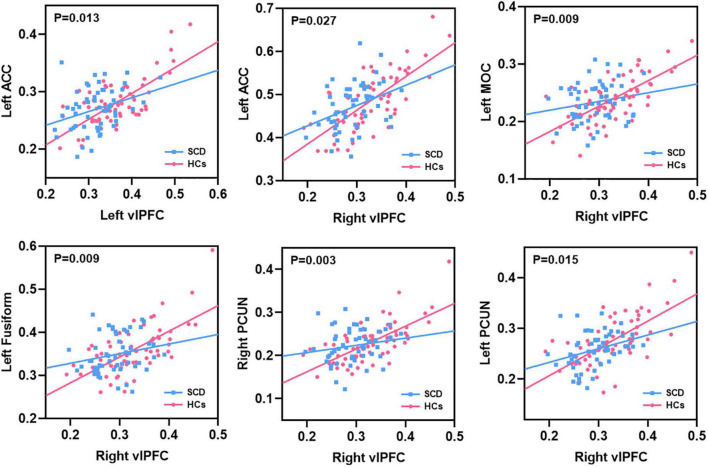
The structural covariance differences between subjective cognitive decline (SCD) patients and healthy controls (HCs). Specific regions showed significant between-group differences with the bilateral vlPFC.

After interaction linear model analysis, the structural covariance differences of bilateral vlPFC were still found to be significantly related to the left ACC, left MOC, left fusiform, and bilateral PCUN (Figure 6).

## Discussion

In this study, we investigated altered PerAF, GMV, and structural covariance patterns in patients with SCD, and the main findings are described below. (1) Patients with SCD had higher PerAF primarily in the right HIPP and right THA; (2) patients with SCD showed significantly decreased GMV in the bilateral vlPFC and right insula; (3) For the left vlPFC ROI, patients with SCD showed decreased structural association with the left ACC, right vlPFC, left insula and right dlPFC. For the right vlPFC ROI, patients with SCD showed decreased structural association with the left ACC, left MOC, bilateral fusiform, and bilateral PCUN. For the right insula ROI, decreased structural covariance was observed between the right insula and regions including right MCC, right PCUN, right MOC, left HIPP, left THA, and right ACC; (4) We performed a between-group analysis of structural covariance patterns by calculating the slope differences. Results indicated that structural covariance differences of the ROIs were still found to be significantly related to the left ACC, left MOC, left fusiform and bilateral PCUN. (5) There were negative correlations between the MMSE and PerAF in the right HIPP, right ITC, and left THA in patients with SCD; (6) There were positive correlations between the MMSE and GMV in the bilateral vlPFC, left STC, and right insula in patients with SCD.

We observed that compared with HCs, patients with SCD had higher PerAF in the right HIPP and right THA, which might indicate a possible compensatory mechanism in the early stage of AD. This is consistent with the ones of previous ALFF-related studies of SCD (Sun et al., 2016). Parker et al. (2020) have also reported that greater areas of increased functional connectivity included the right HIPP, bilateral THA, right posterior division of the parahippocampal gyrus, and bilateral precuneus cortex in patients with SCD relative to HCs. This could reflect a neurocognitive scaffolding mechanism for maintaining stable internal mentation and memory functioning (Park and Reuter-Lorenz, 2009). However, our study found negative correlations between the MMSE and PerAF in the right HIPP, right ITC, and left THA in patients with SCD, indicating that decline in cognitive function is associated with increased PerAF. Increased processing demands for internal mentation and memory at rest may arise due to noisier information traveling through memory systems (Morcom and Henson, 2018). Increasing regional metabolic demands from processing noisy information may serve as a mechanism that induces further neurodegenerative decline. Eventually, this neurodegenerative pressure may shift elevated activity to decreased activity (Viviano and Damoiseaux, 2020).

In this study, patients with SCD showed decreased GMV in the bilateral vlPFC and right insula compared to HCs, which confirmed prior findings of atrophy in the frontal lobe (Hong et al., 2015; Dong et al., 2019). In addition, there were positive correlations between the MMSE and GMV in the bilateral vlPFC, left STC, and right insula in patients with SCD. GMV is associated with cognitive dysfunction in patients with SCD (Dong et al., 2019). The frontal lobe supports high-level cognition comprising executive skills and working memory that is crucial for daily life functioning (Stretton and Thompson, 2012). As a part of the frontal lobe, the vlPFC is associated with working memory (Wang et al., 2019; Li et al., 2021) and there is some evidence that the left vlPFC works in resolving interference from previous items stored in working memory, and the left vlPFC is also reported to have a role in the post-retrieval selection of episodic memory (Thompson-Schill et al., 2002; Weintraub-Brevda and Chua, 2019; Yang et al., 2021). And the right vlPFC is involved in monitoring processes accompanying retrieval. Previous studies have shown that impaired monitoring processes allowing inferences about one’s own memory performance are primarily related to decreasing GMV in vlPFC in patients with AD (Genon et al., 2016). Moreover, the insula is also associated with working memory. Therefore, the decreased GMV in the bilateral vlPFC and right insula may suggest an impairment of memory function and monitoring processes in patients with SCD.

For the left vlPFC ROI, after the interaction linear model analysis, it was still found that the structural covariance pattern of SCD was reduced in the left ACC. Neuroimaging research has indicated that the ACC plays a pivotal role in cognition and attention (Bush et al., 2000; Vázquez et al., 2020), and Wu et al. have also concluded that the ACC neuronal activity correlates with sustained attention (Wu et al., 2017). In previous studies, the ACC has been consistently associated with verbal memory retrieval (Bremner et al., 2004). Additionally, Lesion studies have also found that the ACC is required for learning instrumental tasks (Aly-Mahmoud et al., 2017) and is active during decision-making (Kolling et al., 2016). Decreased structural covariance pattern may indicate that structural association is weakened. It may be that neurodegeneration occurs in some regions and then spreads to other regions (Qing et al., 2021).

For the right vlPFC ROI, after the interaction linear model analysis, it was still found that the structural covariance pattern of SCD was reduced in the left ACC, bilateral PCUN, left MOC and left fusiform. The PCUN, as a crucial node of the DMN, has long been known for a pivotal role in regulating cognitive function. The PCUN demonstrated to be involved in a wide range of brain cognitive functions, such as episodic memory and visuospatial processing (Cavanna and Trimble, 2006). A series of studies have confirmed that the PCUN, together with the interlinked cingulate and prefrontal cortices, has been selectively implicated in episodic memory retrieval-related tasks (Lundstrom et al., 2005). Likewise, an fMRI episodic retrieval study by Henson et al. (1999) has further supported the hypothesis that PCUN activation might reflect the reinstatement of visual images associated with remembered words. Fusiform is an important brain area related to facial cognition (Cai et al., 2015), in fact, the region is mainly implicated in the processing of memory (Köhler et al., 1998) except for facial processing. MOC is located in the primary visual cortex and also plays a critical role in visual cognition and sensory memory-based visual change detecting system (Urakawa et al., 2010). Based on the above evidence, it may be speculated that the abnormal structural covariance of vlPFC with the regions, including the ACC, PCUN, fusiform, and MOC may affect cognitive function to some extent, reflecting decreased ability in memory retrieval, attention, and facial recognition in patients SCD.

However, the affected brain regions were not the same regions by using the PerAF and GMV/structural covariance in the present study. It may be that inconsistency is caused by the different analysis methods. The PerAF is employed to measure brain regional activity by detecting regional signal alterations of spontaneous activity, while the GMV/structural covariance is designed to estimate the gray matter volume size and coordinated variation between different brain regions related to GMV. Different aspects are measured by the methods, and thus, it is not surprising that the affected regions are not located in the same brain regions.

It is notable that although we observed significant differences between groups in MMSE score, the performance of each patients with SCD on the MMSE was still within the age-adjusted normal range. This finding was consistent with the notion that minor subthreshold cognitive dysfunction may be present in patients with SCD (Jessen et al., 2007). In such cases, patients with SCDs are likely to adopt compensatory strategies, including increased functional activity in several brain regions, in order to maintain normal performance in cognitive function in the early stages of the disease. And the findings of our study are consistent with this speculation. Our study found negative correlations between the MMSE and PerAF in the right HIPP, right ITC, and left THA in patients with SCD. There is also a possible explanation for this finding that the neural compensation maintains the impaired neural reserve and then recruits alternate neural networks to improve function (Steffener and Stern, 2012; Mazzeo et al., 2019).

There were limitations in this study. Firstly, our current findings were drawn from a small sample, and larger samples are needed in future studies. Second, the absence of a more detailed neuropsychological assessment should receive attention in the future. Besides, the increased PerAF might be related to the period of SCD, our findings might be due to a performance of the early stages of SCD, and no conclusion can be drawn for the whole SCD. In the future, the early and late stages of SCD can be studied separately to further validate the findings.

## Conclusion

In this study, we detected the increased PerAF in the right HIPP and right THA and decreased GMV in the bilateral vlPFC and right insula. Moreover, the two groups exhibited significantly different structural covariance patterns between the bilateral vlPFC and regions mainly including the left ACC, bilateral PCUN, left fusiform, and left MOC. These findings may imply that patients with SCD have abnormalities of brain structure and function that are implicated in memory retrieval, monitoring processes, attention, and facial recognition. The findings of these results may be the contribution of more novel and reliable methods. Our findings may shed new light on the neuroimaging biomarker of SCD.

## Data availability statement

The original contributions presented in this study are included in the article/supplementary material, further inquiries can be directed to the corresponding authors.

## Ethics statement

The studies involving human participants were reviewed and approved by Ethics Committee, First Affiliated Hospital, Guangxi University of Chinese Medicine. The patients/participants provided their written informed consent to participate in this study.

## Author contributions

PLi and DD were responsible for the study concept and design. YW, LZ, WM, and LL contributed to acquisition of MRI data. KX, SZ, BG, PLu, DC, and XZ assisted with data analysis and interpretation of findings. PLi and KX drafted the manuscript. All authors critically reviewed the content and approved the final version for publication and contributed to the article and approved the submitted version.

## Conflict of interest

The authors declare that the research was conducted in the absence of any commercial or financial relationships that could be construed as a potential conflict of interest.

## Publisher’s note

All claims expressed in this article are solely those of the authors and do not necessarily represent those of their affiliated organizations, or those of the publisher, the editors and the reviewers. Any product that may be evaluated in this article, or claim that may be made by its manufacturer, is not guaranteed or endorsed by the publisher.

## Footnotes

1
http://www.fil.ion.ucl.ac.uk/spm

2
http://rfmri.org/dparsf

3
http://rfmri.org/dpabi
